# Transvaginal Natural Orifice Transluminal Endoscopic Surgery for Surgical Staging of Early-Stage Ovarian Cancers: A Report of Two Cases

**DOI:** 10.3389/fsurg.2022.833126

**Published:** 2022-03-16

**Authors:** Yannick Hurni, Fabien Romito, Daniela Huber

**Affiliations:** ^1^Department of Pediatrics, Gynecology and Obstetrics, Geneva University Hospitals, Geneva, Switzerland; ^2^Department of Gynecology and Obstetrics, Valais Hospital, Sion, Switzerland

**Keywords:** omentectomy, ovarian cancer, natural orifice transluminal endoscopic surgery, surgical staging, vNOTES

## Abstract

Surgical staging is essential in the management of ovarian cancers. This staging has traditionally been performed by laparotomy, but minimally invasive techniques are increasingly employed. Transvaginal natural orifice transluminal endoscopic surgery (vNOTES) is a promising technique in the field of gynecological oncology. We report 2 cases of vNOTES surgical staging for suspicious ovarian tumors. We operated on 2 patients aged of 81 and 62 years for low-grade serous ovarian carcinoma and ovarian cystadenofibroma, respectively. We performed surgical staging with a pure vNOTES technique for the first patient and used a hybrid approach for the second. No intraoperative or postoperative complications were observed. We suggest that vNOTES is a feasible and effective approach to surgically manage early-stage ovarian cancers.

## Introduction

Surgical staging is a crucial part of the early management of suspicious ovarian tumors that is essential to determine a treatment plan and patient prognosis. Surgical staging for ovarian malignancies includes careful peritoneal evaluation; peritoneal washing for cytology; total hysterectomy and bilateral salpingo-oophorectomy; multiple peritoneal biopsies; and resection of any suspicious lesions, infracolic omentectomy, and eventual pelvic and paraaortic lymphadenectomy (1). This staging has traditionally been performed by laparotomy, but minimally invasive techniques are increasingly employed. These include conventional laparoscopy, laparo-endoscopic single-site, robotic multiport, and robotic laparo-endoscopic single-site staging (2–5). Nowadays, increasing evidence supports the safety of these minimally invasive procedures in ovarian malignancy management (5–9), suggesting their efficacy even in performing fertility-sparing surgeries or treating advanced stages in carefully selected patients (10, 11). The role of minimally invasive surgery in the field of gynecology is continuously evolving thanks to the development of new promising techniques such as robotic and natural orifice transluminal endoscopic surgery (12–14).

Transvaginal natural orifice transluminal endoscopic surgery (vNOTES) is an innovative technique permitting access to the peritoneal cavity through the vagina. In recent years, vNOTES has been increasingly applied to perform benign gynecologic procedures such as hysterectomy, myomectomy, several adnexal procedures, uterosacral ligament suspension, and sacrocolpopexy (14–16). To date, the role of vNOTES in gynecological oncology still requires evaluation, as only a few studies have reported on it (17–23).

We present our initial experience with ovarian cancer staging using vNOTES and discuss its feasibility and potential role in managing suspicious adnexal masses.

## Case Description

We report the cases of 2 women who underwent vNOTES surgical staging for suspicious ovarian tumors at Valais Hospital (Switzerland) in October 2021 (Table 1). Both surgeries were performed by the same surgical team: D.H. (principal surgeon) and F.R. (assistant). Patients gave written consent to participate in this study. No formal institutional review board approval was required.

**Table 1:** Demographic, clinical, intraoperative, and postoperative data.

**Case**	**Age (y)**	**BMI (Kg/m^**2**^)**	**Parity**	**Previous abdominal/pelvic surgery**	**Indication for surgery**	**Type of intervention**	**Operative time (min)**	**Blood loss (mL)**	**Length of stay (d)**	**Final pathology**
1	81	22.7	4	CS + tubal sterilization; laparotomic right hemicolectomy and ileal resection with primary anastomosis	Suspicious bilateral adnexal masses on MRI (right 4.1 × 4.2 cm; left 3.1 ×1.4 cm), hypermetabolic on PET/CT CA-125 32 U/mL; CEA 2.6 ng/mL; CA 15–3 28 U/mL; CA 19–9 8 U/mL	TVH + vNOTES bilateral salpingo-oophorectomy, infracolic omentectomy, right pelvic sidewall peritoneal excision, omental appendix lesion excision, peritoneal washing, and multiple biopsies	131	100	4	Low-grade serous carcinoma
2	62	16.9	3	Laparotomic appendectomy	Multilocular cystic right adnexal mass at US exam (max diameter 17 cm) The patient refused any additional exam	Hybrid vNOTES right salpingo-oophorectomy, infracolic omentectomy, peritoneal washing and multiple biopsies	97	30	2	Benign fibrous cystadenoma

*BMI, body mass index; CS, cesarean section; MRI, magnetic resonance imaging; PET/CT, positron emission tomography/computed tomography; TVH, total vaginal hysterectomy; vNOTES, transvaginal natural orifice transluminal endoscopic surgery; US, ultrasound*.

### Surgical Technique–vNOTES Surgical Staging

Patients were placed in dorsal lithotomy position under general anesthesia and received prophylactic intravenous antibiotic with cefuroxime 1.5 g and metronidazole 500 mg. We used a GelPOINT V-Path Transvaginal Access Platform (Applied Medical, Rancho Santa Margarita, CA, USA) as a vNOTES port. We created a pneumoperitoneum to a pressure of 12 mmHg. The operating table was tilted to 20° Trendelenburg position. We used 3 trocars to insert a 10-mm rigid 30° camera and 5-mm instruments such as Johan and bipolar graspers, cold scissors, and a Caiman^®^ grasper. After peritoneal washing was collected, we carefully inspected the adnexes and all peritoneal surfaces to check for eventual extraovarian tumor spread. We performed uni- or bilateral salpingo-oophorectomy and eventual hysterectomy in accordance with each case's specifics. We realized multiple peritoneal biopsies and resected any suspicious lesions. To complete the staging, we performed infracolic omentectomy with the Caiman^®^ grasper. At the end of the procedure, we closed the colpotomy with a running suture using a Stratafix Spiral PDS 0. Clindamycin vaginal cream was administered once a day for the first 7 postoperative days.

### Case 1

**Figure 1:**
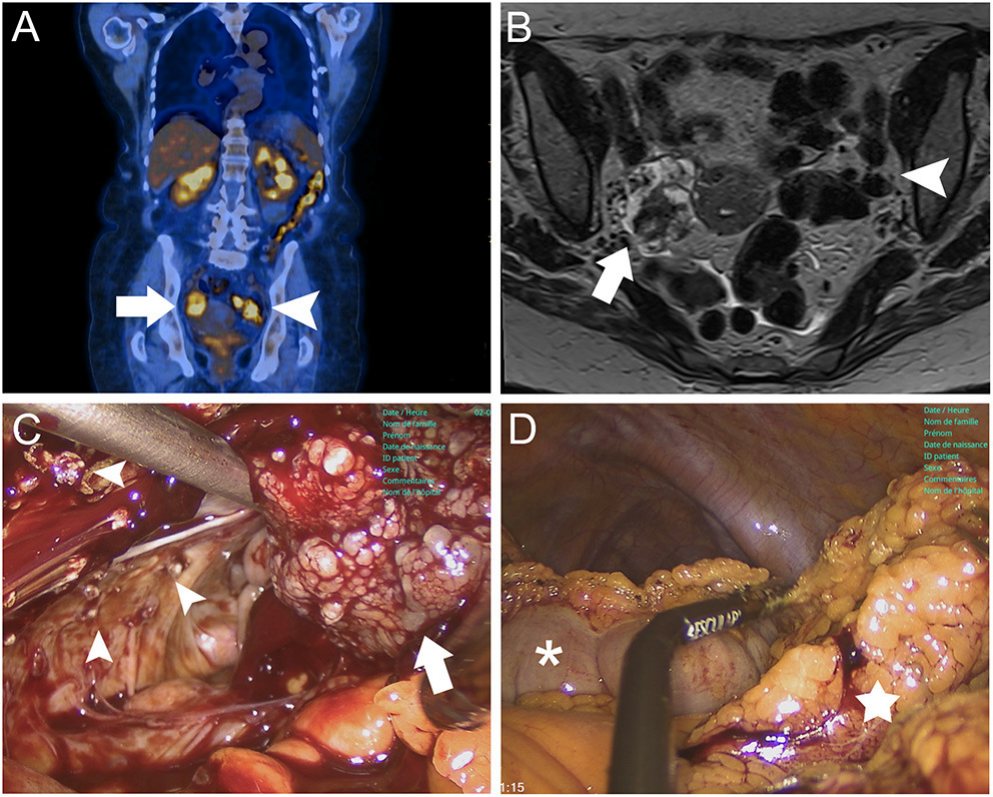
Case 1 images. **(A)** Coronal PET/CT scan demonstrating metabolically active lesions on right (white arrow) and left ovaries (white arrowhead). **(B)** Axial gadolinium-enhanced T1-weighted MRI showing bilateral adnexal masses with heterogeneous solid appearance, irregular contours, and measuring 4.1 × 2.9 × 4.2 cm on the right (white arrow) and 3.1 × 1.4 × 1.0 cm on the left side (white arrowhead). **(C)** vNOTES approach showing a slightly enlarged right ovary with external vegetations (white arrow) and multiple peritoneal implants on the right pelvic sidewall (white arrowheads). **(D)** vNOTES infracolic omentectomy with separation of the greater omentum (white star) from the transverse colon (white asterisk) using a 5-mm Caiman^®^ grasper.

An 81-year-old woman was referred to our service for suspicious adnexal masses. She had recently undergone a laparotomic right hemicolectomy and ileal resection with primary anastomosis for a diffuse large b-cell lymphoma. During postoperative assessments, a positron emission tomography/computed tomography (PET/CT) scan showed suspicious hypermetabolism of both ovaries (Figure 1A). Magnetic resonance (Figure 1B) and sonographic imaging revealed bilateral adnexal irregular solid tumors measuring 4.1 × 2.9 × 4.2 cm on the right and 3.1 × 1.4 × 1.0 cm on the left side. Preoperative PET/CT and magnetic resonance imaging revealed no suspicious lymphadenopathy or additional pulmonary pathology. We decided to perform a surgical staging for these suspicious lesions.

Following a vaginal interadnexial total hysterectomy of a normal-appearing uterus, we completed the intervention using the vNOTES approach. The right ovary appeared slightly enlarged with external vegetations (Figure 1C), while the left adnexa had a normal aspect. On the right pelvic sidewall, we observed multiple suspicious peritoneal implants (Figure 1C) and an adherent omental appendix of suspicious appearance. We performed a bilateral salpingo-oophorectomy; upon extemporaneous pathological examination, we suspected a borderline serous tumor. We decided to complete the surgical staging with infracolic omentectomy (Figure 1D), extended right pelvic sidewall peritoneal excision and omental appendix lesion removal. The intervention lasted 131 min and we observed no intraoperative complications. The patient was discharged on postoperative day 3. No complications were observed within 30 postoperative days, and the patient was satisfied with the surgery. Definitive histopathologic examination revealed a low-grade serous carcinoma of the right ovary (stage FIGO IIB) and a serous cystadenoma of the left ovary. The patient refused adjuvant chemotherapy with carboplatin and taxol. She is receiving hormonotherapy with an aromatase inhibitor (letrozole). Oncology follow-up visits have been planned with CA-125 measurements every 3 months and computed tomography scans every 6 months to monitor both ovarian cancer and large b-cell lymphoma.

### Case 2

**Figure 2:**
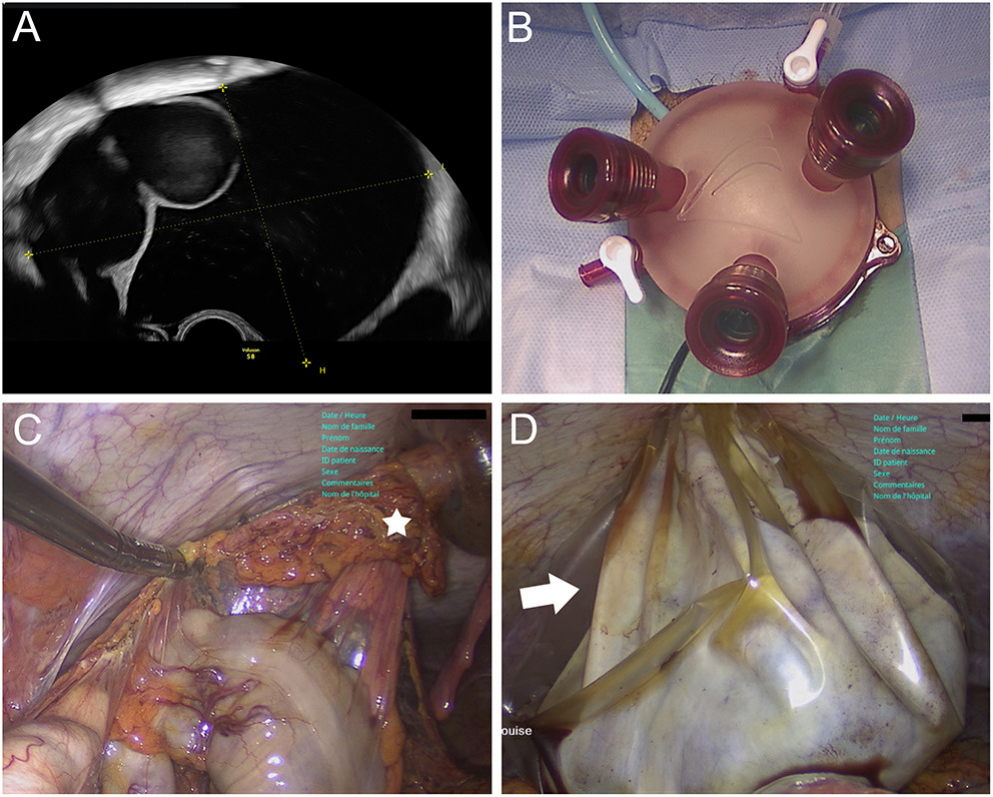
Case 2 images. **(A)** Transabdominal ultrasound showing a smooth multilocular cyst (max diameter 17 cm) originating from right adnexa. **(B)** Installation of the GelPOINT V-Path Transvaginal Access Platform for vNOTES. **(C)** Hybrid vNOTES infracolic omentectomy with removal of greater omentum (white star) from 12-mm supraumbilical trocar. **(D)** Hybrid vNOTES approach with transvaginal view of transabdominal extraction of right adnexal mass into endobag (white arrow).

A 62-year-old woman presented with a history of several months of increasing abdominal distension. Ultrasound revealed a smooth multilocular cyst with greatest diameter of 17 cm originating from the right adnexa (Figure 2A). The patient refused any additional radiologic and blood exam, but consented to undergo surgical staging of this suspicious lesion. We decided to perform the intervention with a vNOTES approach (Figure 2B). The right ovary presented a large cystic mass with a thin wall and no superficial lesions. The uterus and contralateral adnexa appeared normal, and no suspicious lesions were observed on the peritoneum or other intrabdominal organs. Following the abdominal inspection and peritoneal washing, we realized a right salpingo-oophorectomy and infracolic omentectomy (Figure 2C). To limit the risk of involuntary spillage and abdominal contamination, were extracted the specimens into an endobag from a transombilical incision under a continuous transvaginal endoscopic view (Figure 2D). In accordance with the patient's requests, we spared the uterus and contralateral adnexa. The surgery lasted 97 min, and we observed no intra- or postoperative complications. The patient was discharged the first postoperative day. No complications were observed within 30 postoperative days, and definitive histopathologic examination showed a benign cystadenofibroma confined to the right ovary. The patient appeared satisfied with the treatment outcome and no additional visits were planned.

## Discussion

Surgical staging of ovarian cancers has traditionally been performed by laparotomic approach. Since the early 1990s, the role of minimally invasive surgery in gynecological oncology has progressively developed, with conventional and robot-assisted laparoscopic techniques increasingly used to stage and treat endometrial, cervical, and ovarian malignancies (7–9, 24).

In this study, we reported our first experience with vNOTES in ovarian cancer staging. In Case 1, we realized a vaginal total hysterectomy followed by a pure vNOTES staging. In Case 2, the intervention was performed using a hybrid vNOTES approach, allowing transvaginal and transabdominal endoscopic access. These techniques appeared feasible, allowing us to perform the required procedural steps in compliance with oncological principles (1). Both patients achieved full staging without conversion to open surgery. We did not encounter any specific surgical difficulties, and no intra- or postoperative complications were observed. Although lymphadenectomy was not performed, even this challenging surgical step might be feasible using vNOTES. Two techniques have been reported to perform vNOTES lymphadenectomy for the treatment of endometrial cancer using transperitoneal and retroperitoneal approaches (19, 20). Especially the latter appears as a promising method extendable for the treatment of ovarian cancer.

To our knowledge, vNOTES surgical staging for ovarian cancers has been reported only once in the literature (17). Our 2 cases, in addition with the 5 cases reported by Lowenstein et al. (17), suggest that vNOTES might be a feasible and promising technique for surgical management of early-stage ovarian cancers. However, adequate training is essential to benefit from this technique. The learning curve analysis of vNOTES hysterectomy reported by Wang et al. (25) suggests that about 100 vNOTES interventions are needed before complex procedures such as ovarian cancer surgical staging can be properly managed. This is approximately the number of vNOTES operations performed by our surgical team before starting with this procedure.

Compared to open surgery, minimally invasive procedures seem associated with better perioperative outcomes in the management of early-stage ovarian cancers (26). To further reduce the morbidity and scarring related to transabdominal surgeries, ovarian cancer staging by vNOTES was recently introduced at our institution. Despite the limited number of comparative studies, the main advantages of vNOTES over conventional laparoscopy appear to be reduced blood loss, less postoperative pain, lower rate of wound site infection, absence of visible scars, and shorter hospitalization length (14, 27). The transvaginal approach reduces surgical risks associated with (blind) transabdominal peritoneal cavity access, which is mostly important in multioperated patients (see Case 1). Hybrid approaches combine the advantages of different visual and instrumental access methods, allowing endoscopic management of large lesions. In Case 2, a hybrid vNOTES approach allowed safe peritoneal cavity access and secure salpingo-oophorectomy, avoiding the risk of eventual tumor cell spilling. Since vNOTES can be realized with a low-pressure pneumoperitoneum and a steeper Trendelenburg position is rarely needed (18, 27, 28), a reduced impact on cardiovascular and respiratory functions during surgery is observed, making this technique suitable for elderly, obese, and other fragile patients. The main limitations of vNOTES are reduced instrument triangulation, restricted space for manipulations, and limited accessibility of some anatomical regions. In ovarian surgical cancer staging, this is particularly relevant for the inspection of regions that are difficult to view such as the prevesical peritoneum, costodiaphragmatic recesses, Morrison's pouch, and intestinal mesentery, and to perform surgical procedures outside the pelvis such as infracolic omentectomy. The use of articulating instruments and variable-view rigid endoscopes can help overcome these constraints (29). vNOTES appears feasible for cases with no peritoneal carcinosis or when limited to the pelvis, but firm adnexal lesions and the infiltration of nearby pelvic organs such as the rectum could represent an additional limitation of this technique. In the case of extensive disease, the surgical management should be completed with a laparotomic approach.

In conclusion, vNOTES is a feasible and effective technique for surgical staging in early-stage ovarian cancer. More studies are needed to evaluate the long-term oncological outcomes and to appraise the advantages of vNOTES in the general population and for selected cases.

## Data Availability Statement

The original contributions presented in the study are included in the article/supplementary material, further inquiries can be directed to the corresponding author.

## Ethics Statement

Ethical review and approval was not required for the study on human participants in accordance with the local legislation and institutional requirements. The patients/participants provided their written informed consent to participate in this study.

## Author Contributions

DH: proposer of this surgical approach. YH: writing original draft and graphic making. All authors writing review and editing. All authors contributed to the article and approved the submitted version.

## Conflict of Interest

The authors declare that the research was conducted in the absence of any commercial or financial relationships that could be construed as a potential conflict of interest.

## Publisher's Note

All claims expressed in this article are solely those of the authors and do not necessarily represent those of their affiliated organizations, or those of the publisher, the editors and the reviewers. Any product that may be evaluated in this article, or claim that may be made by its manufacturer, is not guaranteed or endorsed by the publisher.
